# Chikungunya virus seroprevalence among febrile patients in selected health facilities in Mount Elgon region, Kenya

**DOI:** 10.1371/journal.pntd.0012169

**Published:** 2025-12-30

**Authors:** Sheila Kageha, Joyce Mwongeli Ngoi, Toru Kubo, Kouichi Morita, Moses Matilu Mwau

**Affiliations:** 1 Kenya Medical Research Institute, Nairobi, Kenya; 2 University of Ghana College of Basic and Applied Sciences, Centre for Cell Biology of Infectious Pathogens (WACCBIP), Accra, Ghana; 3 Nagasaki University, Institute of Tropical Medicine, Nagasaki, Japan; University of Ghana College of Health Sciences, GHANA

## Abstract

**Background:**

Chikungunya is an emerging vector-borne disease of global significance, known for causing acute illness characterized by fever and severe arthralgia, which can evolve to chronic arthritis and rheumatism especially in elderly patients. While febrile illness and arthralgia are common clinical presentations among residents of Mount Elgon (Mt. Elgon), the role of chikungunya virus as a causative agent is undocumented. This study aimed to assess the prevalence of antibodies against Chikungunya Virus (CHIKV) antigens in patients presenting with acute febrile illnesses (AFI) in Mt. Elgon region, Kenya.

**Methods:**

This cross-sectional seroprevalence study involved febrile patients visiting selected health facilities in the region. Serum samples were collected and screened for 1gG + IgM + IgA antibodies using an indirect Enzyme Linked Immunosorbent Assay (ELISA). Positive samples were further analyzed using standard plaque reduction neutralization assay (PRNT) on Vero (Biken) E6 cell cultures for confirmation of presence of neutralizing antibodies against CHIKV.

**Results:**

ELISA results indicated that 317/1359 (23.33%) serum samples were positive for CHIKV antibodies. Of these, 305 samples (96.21%) were subjected to PRNT, 127 samples (9.3%) tested positive for CHIKV neutralizing antibodies.

**Conclusion:**

These findings indicate active circulation of CHIKV in Mt. Elgon region. This underscores the need for enhanced surveillance and monitoring strategies to mitigate the risk of potential outbreaks in the future.

## Author summary

Chikungunya Virus (CHIKV) is an emerging mosquito-borne virus that causes a sudden onset of fever and severe joint pain, often leading to long-term arthritis and rheumatism, especially in elderly population. While it is not typically fatal, the disease can significantly impact quality of life and poses a growing public health concern in regions where the virus is circulating. This study assessed the seroprevalence of CHIKV among patients presenting with acute febrile illnesses (AFI) at selected health facilities in the Mt. Elgon region of Kenya. Using a cross-sectional seroprevalence approach, serum samples from patients were screened for CHIKV antibodies using an indirect Enzyme-Linked Immunosorbent Assay (ELISA). A plaque reduction neutralization assay (PRNT) was subsequently performed on samples that tested positive for CHIKV antibodies to identify the presence of neutralizing antibodies. The findings of this study aim to advance understanding of CHIKV’s epidemiology, and its impact on human health, providing important data for public health policy and future research aimed at controlling and preventing disease outbreaks.

## Introduction

Chikungunya virus (CHIKV), an alphavirus from the Togaviridae family [1–4], was first isolated in 1952–53 from the serum of a febrile patient during an outbreak that occurred on the Makonde Plateau in southern Tanzania [5]. Since then, it has spread worldwide, infecting millions of people. CHIKV infection is commonly characterised by acute fever, headaches and painful arthralgia, which can evolve to chronic arthritis and rheumatism, especially in elderly patients [5–7]. These symptoms are often clinically indistinguishable from those caused by other arboviruses such as dengue, Onyong’ nyong’ (ONNV) and West Nile viruses (WNV). Similarities in clinical symptoms often lead to missed opportunities to detect, and consequent underreporting of CHIKV infection in areas endemic to arboviruses [8,9]. CHIKV has emerged as a global public health threat, with cases of neurologic involvement, fulminant hepatitis, and neonatal encephalopathy reported [10,11]. Severe forms of the disease have also been reported in the Réunion island outbreak of 2005–2006 and in Italy [12–14].

Currently, there are no licensed antiviral drugs or vaccines for preventing CHIKV infection. Treatment is mainly supportive, and entails the use of analgesic drugs in combination with non-steroidal anti-inflammatory drugs for symptomatic relief [15–17]. Patients infected with CHIKV mount robust innate and adaptive immune responses that aid in viral clearance and provide protection. Neutralising anti-CHIKV IgG has been shown to persist for at least 21 months [18–20].

CHIKV circulates in a sylvatic cycle between non-human primate reservoir hosts and *Aedes* spp*.* mosquitoes [21]. *Aedes aegypti* is responsible for CHIKV endemicity in the tropical and sub-tropical regions, while *Aedes albopictus* has been associated with spread in temperate areas, including southern Europe, the Caribbean and southern and eastern parts of the USA. The virus is transmitted to human through infectious bites from these mosquitoes [22,23]. There has been increased frequency of CHIKV outbreaks in previously non-endemic regions, attributed to the expansion of mosquito vector species and genetic adaptations of the virus that enhance its transmissibility [24–26].

Originally endemic in Africa, CHIKV has a wide geographic distribution. Reports of human infections have emerged from Angola, the Democratic Republic of Congo, Mozambique, Gabon, Nigeria, Southern Africa, Tanzania, Uganda, and Burundi [27–31]. The virus has also spread to Asia, including India, and to island countries in the Indian Ocean, as well as to Europe and America. In the United States, the virus has been identified in 45 states, with over 2.9 million suspected and confirmed cases, and 296 reported deaths [29,32,33].

The disease was formerly thought to be self-limiting until the Reunion Island outbreaks in 2005–2006, which resulted in several fatalities. Brazil has also experienced fatalities resulting from Chikungunya outbreak [34]. In Kenya, the first outbreaks of CHIKV infection were recorded in Lamu and Mombasa in 2004 [35,36]. Genetic analysis indicated that the virus responsible for the Indian Ocean epidemic originated from the Kenyan Coast [37]. In Kenya, CHIKV has continued to circulate in different parts of the country including Western and Coastal regions [38–42]. According to the World Health Organization, the estimated global seroprevalence of CHIKV is approximately 24%. The highest rates were reported in a study conducted in Laos, while the African region showed the highest regional prevalence, estimated at around 31% [41].

Mt. Elgon region in Trans Nzoia County is ecologically suited for arbovirus transmission due to high vector density, the presence of reservoir hosts, and climatic conditions favorable for virus circulation [43]. Human encroachment into forested areas for agricultural expansion increases exposure to sylvatic mosquito vectors responsible for transmission of these viruses [44,45]. According to the 2019 Kenya Population and Housing Census, the region has an estimated population of over 990,000 and is a gateway to Mt. Elgon National Park, a known tourist destination. This study aimed to assess the seroprevalence of CHIKV infection among febrile patients presenting at selected health facilities in the region.

## Materials and methods

### Ethical considerations

Written informed consent was obtained directly from adult participants. In the case of children, written consent was provided by a parent or guardian, and assent was obtained from minors capable of providing it. To protect participants’ identities, samples and data were anonymized, with unique codes assigned to each participant to ensure privacy. The study protocol was approved by Kenya Medical Research Institute (KEMRI) Ethical Review Committee under protocol number SSC. 1698. A comprehensive data management plan was put in place to ensure secure collection, storage, analysis, and reporting of data in compliance with data protection regulations.

### Study design and study site

This was a cross-sectional study conducted among patients presenting with acute febrile illnesses (AFI) at selected health facilities in Mt. Elgon region, Trans Nzoia County. Mt. Elgon ecosystem comprises a forest reserve, and a national park, providing favorable climatic conditions and a steady supply of reservoir hosts for vector-breeding. This environment increases the risk of exposure to sylvatic mosquitoes that transmit these viruses.

**Fig 1:**
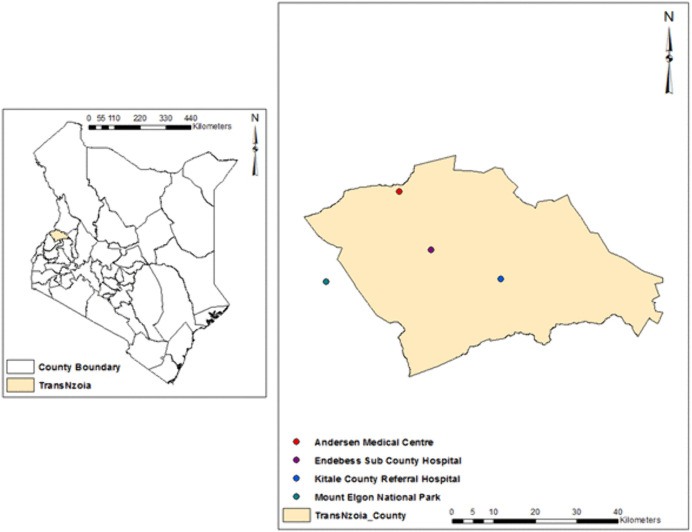
Map showing the hospital-based study sites located in Trans Nzoia County in Kenya where samples were collected. Basic digital administrative boundaries map was free download (open source) from https://gadm.org/maps/KEN/transnzoia.html. The maps were generated using ArcMap 10.2.2 (http://desktop.arcgis.com/en/arcmap) advanced license) courtesy of Samuel Arach Owaka.

The study was strategically carried out at three health facilities: Andersen Medical centre (AMC), Endebes sub-district hospital (END), and Kitale County Referral hospital (KCRH) (Fig 1).

These sites were selected based on their service to a large and demographically diverse population, accessibility, and high reported incidence of febrile illnesses; making it an ideal setting for investigating arboviral seroprevalence.

### Study participants’ recruitment

With written informed consent/assent, a 5-ml venous blood samples were collected from febrile patients ≥ 5 years of age seeking healthcare at the selected health facilities within the region. This was to ensure safer and more practical blood collection, as younger children face higher procedural risks and discomfort. The selected age bracket also represents a more mobile and socially active group, with an increased risk of exposure to mosquito vectors. Studying this population provides valuable insights into the transmission dynamics within the community. Fever was defined as temperature range from a minimum of 38 **°**C to a maximum of 40.5 **°**C. The collected samples were stored at -20 **°**C at the facilities and transported in dry ice for processing at the Kenya Medical Research Institute (KEMRI), where sera were separated, aliquoted into vials and stored at -80 **°**C.

Structured questionnaires were used to gather socio-demographic and clinical information from a subset of study participants. Demographic information, including age, gender, residence, and occupation, as well as clinical information—such as specific signs and symptoms commonly associated with arboviral infections (e.g., headache, rash, joint pain, muscle pain, jaundice)—were collected.

### Antibodies detection methods

The antibody detection methods used in this study included enzyme-linked immunosorbent assay (ELISA) to detect IgG, IgM, and IgA antibodies against CHIKV, as well as plaque reduction neutralization assay (PRNT) to identify neutralizing antibodies against CHIKV antigens in samples that tested positive by ELISA. The ELISA utilized a polyclonal anti-human IgG + IgM + IgA conjugate (American Qualex), which detects total antibody responses rather than isotype-specific reactivity. This approach was selected to maximize sensitivity for detecting CHIKV exposure, particularly in individuals at varying stages of infection.

Initially, all sera were screened for antibodies against CHIKV antigens using an in-house indirect ELISA based on the Igarashi Technical Manual (2000) [46], with some modifications. Antigens used for the indirect ELISA were obtained from purified CHIKV infected C6/36 cell cultures. Virus antigens were concentrated using polyethylene glycol and sodium chloride (NaCl), then purified via sucrose-gradient ultracentrifugation at 50000g, for 14 hours at 4 °C (Bundo and Igarashi, 1983) [47]. The viral antigens were diluted to 250ng/100 µL with phosphate buffered saline (PBS). High- protein-binding 96-well microplates (Maxisorp; Nulgenunc international, Roskilde, Denmark) were coated with 250ng/100 µL/well of viral antigen, wrapped and incubated overnight at 4 °C. Blocking achieved by adding 100 µL of PBS, with 3% FCS (PBS-F) to each well, followed by a 30-minute incubation at room temperature (RT). Test sera were diluted at 1:1000 in PBS-F and added in duplicates to the pre-coated plates, and incubation done at 37 °C for 1 hr. Control sera (both positive and negative) were included on each plate; the positive control sera was obtained from the KEMRI- Viral haemorrhagic fever laboratory. After incubation, the plate was washed three times with PBS, containing 0.05% Tween-20. Then, 100 µL/well of 1:5000 diluted horseradish-conjugated goat anti-human IgG + IgM + IgA (American Qualex, A139PN) [48] was added, followed by another 1-hour incubation at 37^o^C, followed by three washes. A substrate solution containing of 5mg O-Phenylenediamine dihydrochloride (OPD) (Sigma Chemical, St. Louis, MO) was added, and the reaction was incubated for 15 minutes in the dark at RT. 100 µL/well of 5N Sulfuric acid was then added to stop further reaction. The Absorbance (OD) of each well was measured using spectrophotometer at 492nm, with OD specific for CHKV calculated as [(Mean OD of virus-coated wells) – (Mean OD of PBS-F-coated wells)]. A sample was deemed positive if the mean OD difference was ≥ 1.1, negative if <0.8, and borderline if ± 0.2.

For PRNT assay, CHIKV antigens were derived from bulk virus cultures (S27 prototype African strain) and purified using sucrose density gradient ultracentrifugation. Virus stock titres were determined by plaque assays in Vero cells, expressed as plaque‐forming units (PFU) per ml. All patient sera that tested positive for IgG + IgM + IgA ELISA were subjected to PRNT (protocol adapted from Igarashi Technical Manual, 2000). This assay was used to determine the presence of CHIKV specific neutralizing antibodies in the test sera. PRNT was performed using Vero E6 cells. Plates were prepared by seeding Vero cells at a concentration of 1 x 10^5^ cells/ml into 6-well plates at a volume of 3ml/well. The cells were propagated in Eagle’s minimum essential medium (EMEM) (Gibco), 10% fetal calf serum (FCS) (Gibco), L-glutamine P/S (Gibco), 0.2mM NEAA (Gibco), NaHCO3 supplemented for 1 day at 37 °C, 5% CO_2_ until the cells had attained 80% confluence. Test serum was diluted in Maintenance Medium (MM) (EMEM, 2% FCS, L-glutamine, P/S, NEAA, NaHCO3 supplemented) into 1:10 dilutions. At the same time, standard virus dilution of 1000 PFU/ml with MM was prepared. Equal volumes of the serum dilution (100 µL) and standard virus dilution (100 µL) were mixed, with positive control wells containing equal volumes of MM and standard virus. The virus-serum mixture was then incubated for 1 hour at 37 **°**C. After incubation, 2.5 mL of culture medium from each well was aspirated, and 100 µL of the serum/virus mixture was added to Vero cells in duplicate wells. The plates were then incubated for 1.5 - 2 hours at 37 **°**C, 5% CO_2_. Following this, 3 mL of overlay medium (EMEM, 2% FCS, 1.4% Methylcellulose, P/S supplemented) was added to each well, and plates incubated at 37 **°**C, 5% CO_2_ for 2–3 days with daily observations. Harvesting involved discarding of the overlay medium and fixing plates by adding 1 ml of 10% formalin in PBS over the cell and incubating for 1 RT in a safety cabinet under UV light. After incubation, the plates were gently washed with tap water at least twice, absorbed on paper towel and stained with 0.5 ml of 1% Crystal Violet solution in water. The stain was removed after 5–10 minutes at RT, and the plates gently washed with tap water. The plates were air-dried at RT and plaques counted for each set of duplicate wells. The percentage reduction was calculated by comparing with the positive control well (100% plaque formation). Sera were initially tested at a dilution of 1:20, and those that reduced the number of plaques by ≥75% (PRNT_75_) were further titrated. More than 90% plaque reduction (PRNT_90_) was considered positive, with titres expressed as the reciprocal of serum dilutions yielding ≥90% reduction in the number of plaques.

### Data analysis

To assess the seroprevalence of Chikungunya Virus (CHIKV) antibodies among patients, descriptive statistics, which included the mean and median age, and temperature ranges were used to summarize participants’ characteristics. To evaluate potential associations and inferential statistics, Poisson Regression Analysis was used, with two-sided p-values reported. Statistical significance was set at p < 0.05. Data analyses were performed using STATA Version 14 software (Stata Corp LP, College Station, Texas, United States).

## Results

A total of 1,398 samples were collected from febrile patients at the selected health facilities and screened for CHIKV antibodies. Thirty-nine samples (2.79%) were excluded from analysis due to insufficient serum volume and compromised sample integrity. Among the study participants, temperature ranging from a minimum of 38 **°**C to a maximum of 40.5 **°**C, with a mean of 39.1 **°**C were identified. In addition to fever, some patients also exhibited specific signs and symptoms commonly associated with arboviral infections, such as headache, rash, joint pain, and jaundice. Other patients reported muscle pain, general malaise, abdominal pain, diarrhoea and bleeding.

Demographic and clinical data were available for 782 out of 1,359 samples (57.54%): 507 samples (37.31%) were collected from females and 275 samples (20.24%) from males. The mean age of the participants was 31.6 *± 0.57* years (SEM), with a median of 28, and a range of 5–91 years. There was no significant age difference between males and females, (median ages of 31.4 and 31.7, respectively; *p* > 0.6). Information on patient residence was available for 777 samples, with the majority residing in formerly Rift Valley Province (83.71%), followed by Western Province (2.1%), Eastern Uganda (11.2%) while the rest (3%) were non-residents. Of the 1,359 samples processed, 689 (50.70%) were from KCRH, 463 (34.07%) from AMC and 207 (15.23%) from END. A total of 317 samples (23.34%) tested positive for CHIKV antibodies by ELISA.

The odds of exposure were assessed based on gender, health facilities, age of participants and presenting symptoms (Table 1). The odds of being seropositive for CHIKV were significantly lower in END and KCRH compared to AMC, and for those with eye infections and headaches compared to those without these symptoms (p < 0.05) (Table 1).

**Table 1:** Demographic and clinical characteristics of study participants and CHIKV test outcomes (N = 1,359).

	Health Facility			Total n (%)	CHIKV positive by ELISA (n = 317		
Variable	KCHR (n = 689)	AMC (n = 463)	END (n = 207)		OR	95% CI	*p* value
**Gender**							
Male	139	94	42	275 (20.24)	Ref.		
Female	282	161	64	507 (37.31)	0.68	0.45 - 1.04	0.08
Missing data*	268	208	101	577 (42.46)	0.29	0.19 - 0.43	0.00
**Age group**							
0 - 5.0	2	10	0	12 (0.88%)	2.3	0.30 - 1.81	0.43
5.1 - 20	97	44	13	154 (11.33%)	1.08	0.65 - 1.77	0.77
20.1 - 35	201	130	45	376 (27.67%)	Ref.		
35.1 - 50	67	37	24	128 (9.42%)	1.13	0.65 - 1.95	0.67
50.1 - 100	43	29	12	84 (6.18%)	1.14	0.83 - 3.66	0.14
missing data*	279	213	113	605 (44.52%)	0.43	0.31 - 0.59	0.001
**Seropositivity**							
IgM + IgG + IgA	156 (22.64%)	135 (29.16%)	26 (12.56%)	317			
PRNT positive	76 (11.03%)	40 (8.64%)	11 (5.31%)	127			
**Health Facility**							
KCHR				689 (50.70%)	0.72	0.55 - 0.94	0.01
AMC				463 (34.07%)	Ref.		
END				207 (15.23%)	0.35	0.22 - 0.56	0.00
**Presenting symptoms**							
**Rash**							
No				556 (40.91%)	Ref.		
Yes				224 (16.48%)	1.07	0.70 - 1.62	0.76
Missing data*				579 (42.62%)	2.72	2.03 - 3.64	0.00
**Eye Infection**							
No				617 (45.40%)	Ref.		
Yes				159(11.70%)	0.53	0.30 - 0.92	0.023
Missing data*				583 (42.90%)	2.35	1.79 - 3.09	0.00
**Jaundice**							
No				659 (48.49%)	Ref.		
Yes				115 (8.46%)	1.06	0.62 - 1.80	0.84
Missing data*				585 (43.05%)	2.67	2.02 - 3.52	0.00
**Headache**							
No				205 (15.08%)	Ref.		
Yes				568 (41.80%)	0.54	0.36 - 0.82	0.00
Missing data*				586 (43.12%)	1.72	1.19 - 2.50	0.00

This table summarizes the demographic characteristic, clinical presentations and laboratory test outcomes of 1,359 study participants across the three health facilities. Values are presented as counts (n) and their corresponding percentage (%) within the total study population (N = 1,359. The odds ratios (OR), 95% confidence intervals (95% CI) and p-values describe associations with CHIKV seropositivity based on ELIZA results (n = 317). Missing data indicate records with incomplete demographic or clinical information.

KCRH refers to Kitale County Referral Hospital, AMC stands for Andersen Medical Center; and END indicates Endebes sub-district Hospital. Age group categorization began at 0 to facilitate analysis of trends across various age groups; however, study participants were recruited starting from age 5. N = total number of study participants, n = number of participants within each category, % = percentage of participants relative to the total study population (N = 1,359), OR=odds ratio for association with CHIKV ELISA positivity, CI = confidence interval. *missing data represents participants lacking complete demographic or clinical information.

Out of the 317 samples that tested positive for antibodies by ELISA, 305 (96.21%) had sufficient serum volume for further analysis and were subsequently tested by PRNT for CHIKV-specific neutralizing antibodies. Among these ELISA-positive samples, 123 (41%) showed neutralizing activity against CHIKV antigens by PRNT, while the remaining 177 (59%) failed to neutralize. In contrast, among the five ELISA-negative (borderline negative) samples, 4 (80%) were found to be neutralizing by PRNT, demonstrating that ELISA missed a small number of true positives (borderline samples were those with OD levels close to the cut-off for a positive result) (Table 2).

**Table 2:** ELISA reactivity compared with PRNT outcomes for CHIKV (n = 305).

ELISA Status	CHIKV PRNT (n = 305)		
	Neutralizing	Non-Neutralizing	Total
Negative	4	1	5
	(80.0%)	(20.0%)	(100.0%)
Positive	123	177	300
	(41.0%)	(59.0%)	(100.0%)
**Total**	**127**	**178**	**305**
	**(41.64%)**	**(58.36%)**	**(100.0%)**

This table shows the distribution of plaque reduction neutralization test (PRNT) results among ELISA-positive samples for Chikungunya virus (CHIKV). For each ELISA category, the number of samples classified as neutralizing or non-neutralizing by PRNT is presented, along with the corresponding percentages. The final row summarizes the overall distribution of PRNT results across all tested samples.

n = number of samples tested

%=percentage of samples within the row category

ELISA = enzyme-linked immunosorbent assay

PRNT = plaque reduction neutralization test

CHIKV = Chikungunya virus

## Discussion

Our study found an overall seroprevalence of 23.34% (317/1,358; 95% CI:21.1% - 25.6%) based on ELISA testing. PRNT results indicated positive findings in 9.35% (n = 127) of the samples, confirming CHIKV activity in the region. Mt. Elgon area has a rich biodiversity, supported by forests, rivers, high levels of humidity and vertebrate reservoirs that provide potential breeding habitats for *Aedes* spp*.* of mosquitoes. These ecological settings facilitate breeding and the spread of vector-borne viruses in the region. Since agriculture is the main economic activity in the region, contact with vector is likely. The region has experienced encroachment into forest reserves for agriculture and extraction of forest products, including fuelwood, medicinal herbs, timber, grazing and hunting of wild game. This encroachment increases exposure to sylvatic mosquitoes that vector these viruses. Prior studies have identified *Aedes* spp. as the vectors responsible for transmission CHIKV in Western Kenya [49,50].

The observed seropositivity of 23.34% compares with findings from previous studies in East Africa, suggesting that alphaviruses-though often clinically subtle- are actively circulating in the region [51]. A study conducted in Uganda, a neighbouring country, identified CHIKV as one of the causative agents of acute febrile illness (AFI) [52]. In addition, evidence from other African countries, including Sudan, Ethiopia, Tanzania, Cameroon, Benin, Mozambique and Guinea, highlights substantial yet often unrecognized circulation of CHIKV across the continent [53–58].

In this study, we could not ascertain with confidence whether the observed seropositivity was due to current or past CHIKV and/or ONNV infections. There is limited knowledge regarding the lifespan of CHIKV specific antibodies beyond what is generally known about IgG and IgM antibodies kinetics. However, herd immunity in populations with frequent exposure to vectors may account for the observed neutralization [56,59]. Although not originally included in the methodology, we decided to incorporate some of the borderline negative samples to provide further insights into potential low-level/waning or early-stage infections that might not elicit a strong response detectable by ELISA. Four samples that tested negative (borderline) by ELISA were later confirmed positive by PRNT, highlighting the limitations of relying solely on fixed ELISA thresholds and emphasizing the need for careful interpretation of serological data. These findings underscore the importance of confirmatory testing using a combination of diagnostic methods, such as reverse-transcription quantitative polymerase chain reaction (RT-qPCR) or PRNT, the latter which is often considered the gold standard for detecting the presence of neutralizing antibodies. Such an approach provides a more definitive indication of past, present or convalescent infection.

Interestingly, 178 of the 305 samples (58.36%) that tested positive by ELISA did not neutralize the CHIKV antigens by PRNT. This suggests potential cross-reactivity of IgM/IgG with closely related alphaviruses circulating in the region. CHIKV is part of the Semliki Forest virus (SFV) antigenic serocomplex, which exhibits serological cross-reactivity [59,60]. Co-infections with multiple pathogens can complicate diagnosis, severity and management of these viruses [61,62]. Future studies assessing CHKV antibody persistence and potential cross-protection from previous infections could yield valuable insights. Related studies have indicated the presence of multiple genotypes that may influence serological outcomes and epidemic potential [27,63].

In Kenya, particularly in malaria-endemic regions, malaria is often diagnosed as the cause of acute undifferentiated febrile illnesses, while other causes like arboviruses are frequently overlooked. This study suggests that CHIKV may be a significant cause of fever in Mt. Elgon and should be considered in clinical differential diagnosis.

As a baseline study, this research faced limitations, including incomplete or missing demographic and clinical data for a large portion of study samples, which may have introduced bias and limited a more comprehensive exploration of variable interactions and the analysis of exposure odds. However, the primary focus of the analysis was on serological data, where the missing demographic variables did not significantly affect the core findings. A more comprehensive regression approach could be considered in future studies with complete datasets.

This study was conducted within health facilities and may therefore not fully represent the broader population, particularly those individuals who self-treat or seek care outside formal healthcare systems (use alternative medicine). Although we selected accessible and diverse health facilities to capture varied febrile cases, individuals who do not seek treatment from hospitals may have been missed. Future research incorporating community-based surveys are necessary to understand CHIKV transmission dynamics and burden, and to guide public health initiatives. Enhancing data collection techniques, field monitoring, and adequate training for data collectors will help address current data gaps, thereby, improving our understanding of CHIKV infections in the region. Ultimately, the insights gained from both this baseline and future follow-up studies is expected to guide targeted public health interventions and ensure effective resource allocation for the prevention and mitigation of the impacts of CHIKV in the region.

## Conclusion

The findings of this seroprevalence study have significant implications for both public health programs and future CHIKV control efforts. Our study underscores the frequent, yet often overlooked, impact of Chikungunya virus (CHIKV) in Mt. Elgon region, emphasizing the need for regular surveillance to monitor the virus’s transmission and to guide effective public health interventions. Periodic monitoring and follow-up studies would provide valuable insights into the immune response during recovery and help identify key risk factors, which could improve targeted prevention efforts, such as awareness campaigns and resource allocation.

Moreover, the study highlights the importance of strengthening public health systems, particularly through targeted interventions like enhanced vector control, increased community awareness, and improved diagnostic capabilities. Conducting seroprevalence studies in high-risk areas, such as the one in Mt. Elgon, provides a clearer understanding of CHIKV’s spread, helping health authorities develop more effective prevention and control strategies.

In addition, the study highlights the challenges of cross-reactivity in routine diagnostics, advocating for the use of confirmatory tests like RT-qPCR and PRNT to improve the accuracy of CHIKV detection and differentiate it from related viruses.This study therefore, lays a foundation for future efforts to protect vulnerable populations, enhance diagnostic accuracy, and build more resilient public health systems capable of responding to the ongoing and potential future CHIKV outbreaks.

## Supporting information

S1 Dataset De-identified dataset file. **Title**: Chikungunya Virus Seroprevalence Among Febrile Patients in selected health facilities in Mount Elgon region, Kenya. **Blank** = Missing information. **Red** = Sample not processed.
(CSV)
